# Cognitive functions in children with congenital adrenal hyperplasia

**DOI:** 10.20945/2359-3997000000125

**Published:** 2019-04-15

**Authors:** Nermine Hussein Amr, Alaa Youssef Baioumi, Mohamed Nagy Serour, Abdelgawad Khalifa, Nermine Mahmoud Shaker

**Affiliations:** 1 Department of Paediatrics Ain Shams University Cairo Egypt Department of Paediatrics, Ain Shams University, Cairo, Egypt; 2 Department of Paediatrics Ain Shams University Cairo Egypt Department of Paediatrics, Ain Shams University, Cairo, Egypt.; Kent, Surrey and Sussex, Health Education England UK Kent, Surrey and Sussex, Health EducationEngland, UK; 3 Institute of Psychiatry Ain Shams University Cairo Egypt Institute of Psychiatry, Ain Shams University, Cairo, Egypt; 4 Department of Psychiatry Ain Shams University Cairo Egypt Department of Psychiatry, Ain Shams University, Cairo, Egypt

**Keywords:** Congenital adrenal hyperplasia, children, cognitive functions, corticosteroid treatment

## Abstract

**Objective:**

There is controversy regarding cognitive function in patients with congenital adrenal hyperplasia (CAH). This study is aimed at the assessment of cognitive functions in children with CAH, and their relation to hydrocortisone (HC) therapy and testosterone levels.

**Subjects and methods:**

Thirty children with CAH due to 21 hydroxylase deficiency were compared with twenty age- and sex-matched healthy controls. HC daily and cumulative doses were calculated, the socioeconomic standard was assessed, and free testosterone was measured. Cognitive function assessment was performed using the Wechsler Intelligence Scale – Revised for Children and Adults (WISC), the Benton Visual Retention Test, and the Wisconsin Card Sorting Test (WCST).

**Results:**

The mean age (SD) of patients was 10.22 (3.17) years [11 males (36.7%), 19 females (63.3%)]. Mean (SD) HC dose was 15.78 (4.36) mg/m ^2^ /day. Mean (SD) cumulative HC dose 44,689. 9 (26,892.02) mg. Patients had significantly lower scores in all domains of the WISC test, performed significantly worse in some components of the Benton Visual Retention Test, as well as in the Wisconsin Card Sorting Test. There was no significant difference in cognitive performance when patients were subdivided according to daily HC dose (< 10, 10 – 15, > 15 mg/m ^2^ /day). A positive correlation existed between cumulative HC dose and worse results of the Benton test. No correlation existed between free testosterone and any of the three tests.

**Conclusion:**

Patients with CAH are at risk of some cognitive impairment. Hydrocortisone therapy may be implicated. This study highlights the need to assess cognitive functions in CAH.

## INTRODUCTION

Congenital adrenal hyperplasia (CAH) is a group of autosomal recessive diseases, the most common enzyme defect of which is 21-hydroxylase deficiency. This defect is characterised by defective cortisol synthesis, elevation of adrenocorticotrophic hormone, and accumulation of adrenal androgens. Patients present in early life with virilisation of female newborns or by salt-losing crises which can occur in both sexes ( 1 ).

Cognitive functions represent the highest environmentally acquired mental functions of the human brain. The four main areas of cognitive assessment are 1) attention, which is the ability to maintain concentration on a single stimulus in an environment containing other distracters; 2) perception, which is an essential step in processing sensory information; 3) memory; 4) executive functions, which are the most advanced of all cognitive functions as they encompass integration of input from the environment with what is stored in memory to be used for planning and choosing strategies ( 2 ).

Data on cognitive functions in CAH patients is controversial ( 3 ). Visuospatial processing may be affected by prenatal androgen exposure through an effect on the hippocampus ( 4 ). Memory performance defects were demonstrated in some patients with CAH ( 5 ), some showed defects in mental rotation ( 6 ), executive functions ( 7 ), spatial abilities ( 8 ), while others showed no differences in cognitive functions between patients and controls ( 9 ). The androgenic effect on spatial abilities during a critical time of development was thought to be curvilinear, so that very low and very high doses of testosterone impair cognition, whereas doses in the intermediate zone improve cognition ( 4 ). There is paucity of such data in children with CAH ( 5 , 10 , 11 ). Cognitive deficits are hypothesized to result either from the disease process itself owing to prolonged exposure to high androgen levels, or could be the effect of glucocorticoid medication that is used in the treatment ( 3 , 4 ). Another postulated mechanism is the effect of hyponatraemia ( 4 ).

The aim of this study is to assess the cognitive functions in children with CAH, and their relation to hydrocortisone (HC) therapy and testosterone level.

## SUBJECTS AND METHODS

Thirty patients aged 6-16 years with classic congenital adrenal hyperplasia due to 21 hydroxylase deficiency were included in this case–control study. Patients were recruited during between August 2014 and December 2015 from the Pediatric Endocrinology Clinic at Ain-Shams University Hospital, Cairo, Egypt. All parents signed an informed consent prior to recruitment and the patients dis so as well if this was deemed appropriate. The study protocol was approved by the local ethics committee of Ain-Shams University. Diagnosis was made based on clinical signs and biochemical assessment (elevated ACTH, 17 hydroxy-progesterone, dehydroepiandrosterone, androstenedione, total and free testosterone, in addition to low cortisol). Salt-wasting (SW) was diagnosed in patients with frank hyponatraemia and hyperkalaemia accompanied by low plasma aldosterone and elevated renin concentrations. All patients were receiving glucocorticoid treatment in the form of hydrocortisone. No antenatal dexamethasone was used in any of the patients. Salt-wasting patients were on 9-alphafludrocortisone therapy at a dose of 50–100 µg/m ^2^ /day. The simple virilising (SV) patients did not receive mineralocorticoid treatment. Patients were excluded from the study if they had other chronic medical conditions or co-morbid psychiatric diagnoses.Twenty age- and sex-matched controls were recruited from the General Pediatric Outpatient Clinic of Ain-Shams Children’s University Hospital. Controls were healthy subjects attending the outpatient clinic accompanying their sick siblings.

### Clinical evaluation

Patients’ files were reviewed for date of birth, age at diagnosis, ambiguity at birth, sex assignment, and karyotype. Family history of similar condition was also reported.

The mean daily dose of hydrocortisone in relation to body surface area was calculated and patients were subdivided according to their daily dose (< 10 mg/m ^2^ /day, 10-15 mg/m ^2^ /day, > 15 mg/m ^2^ /day) at the time of assessment. Duration of treatment and total hydrocortisone dose since the start of treatment was calculated in milligrams. All patients and controls underwent physical examination. Height was measured without shoes to the nearest 0.1 cm, using the Harpenden stadiometer (Holtain ltd, Croswell, Crymych, UK). Weight was measured using a digital scale, to the nearest 0.1 kg, wearing light clothing and without shoes. Body mass index (BMI) was calculated using the formula kg/m ^2^ . Standard deviation scores for weight, height, and BMI were calculated ( 12 , 13 ). Socioeconomic status (SES) was determined for patients and controls based on parental education and occupation, family income, crowding index, and sanitation available. A score of 25–30 is considered high SES, 20–24 middle, 15–19 score low SES, and < 14 is very low SES ( 14 ). Patients showed different pubertal stages ranging from Tanner stage 1 to Tanner stage 5 in both males and females. Patients were not categorised according to their pubertal stage to detect the effect puberty might have on cognitive function, as this would have created many subgroups in male and female patients which would have decreased the individual group power when trying to correlate the resulting subgroups with their various cognitive function tests. However, cognitive function tests were applied and comparison was done to age and sex-matched controls.

### Laboratory evaluation

Serum free testosterone level was measured for patients only, as part of a routine assessment with a commercially available kit (DRG International Inc., USA, EIA-2924) using the principle of competitive immunoenzymatic colorimetric assay.

### Neurocognitive functions assessment

All patients and controls underwent the following tests.

#### Wechsler Intelligence Scale-Revised for Children and Adults (WISC)

WISC reflects the intellectual performance through verbal (VIQ), performance (PIQ), and full scale IQ. It is used for assessment of different cognitive functions, including executive functions, and provides an overview of the integrity of cognitive abilities ( 2 ) and its Arabic version has been validated ( 15 ). The WISC comprises ten subtests numbered in order of their administration, in which verbal and performance tests are alternated. The Wechsler Intelligence Scale for Children assesses a panel of cognitive function parameters. Five verbal linguistic subtests underlie the verbal IQ; information (factual knowledge), similarities (verbal concept formation), arithmetic (mental arithmetic), vocabulary (word definitions), and comprehension (social understanding). Five visuospatial subtests underlie the performance IQ: picture completion (perception of visual detail), picture arrangement (logical reasoning), block design (visual analysis), object assembly (part/whole construction), and coding (symbol manipulation). The test also measures the total IQ. This test is also a practical tests of the working memory. Scoring is as follows: > 90 is normal, 90-70 borderline, and < 70 is borderline. VIQ and PIQ were interpreted as normal if > 7 in each subscale, abnormal if < 7 ( 16 ).

#### Benton Visual Retention Test

This test assesses visual perception, visual memory and visuoconstructive abilities. It measures perception of spatial relations and memory for newly-learned material. The examiner compares the examinee’s obtained scores with the expected scores found in the norm tables. The larger the difference, the more probable it is that the examinee has neurological impairment. A difference <4 is a normal score ( 17 ).

#### Wisconsin Card Sorting Test (WCST)

WCST is a tool for recognising frontal cortical executive functions (planning – shifting – cognitive flexibility – sustained attention) ( 18 ). A computer-based test is used, in which 4 stimulus cards appear on the screen, with symbols differing in colour, shape, and number. A fifth card is presented to the child, and the child is asked to match the card presented with one of the 4 stimulus cards and sort it under the most suitable stimulus card. The examiner declares if this match is right or wrong and accordingly the child keeps or changes his chosen strategy. Ten indices are chosen for assessment (total trials administered, total correct trials, total errors, percentage errors, trials to complete first category, failure to maintain set, percentage conceptual level response, learning to learn, categories completed, and percentage preservative). The test is normal if the number of categories completed >6 ( 18 ).

## Statistical analysis

IBM SPSS Statistics (V. 22.0, IBM Corp., USA, 2013) was used for data analysis. Data is expressed as mean & standard deviation (SD) for parametric values, median and interquartile range (IQR) or 25 ^th^ –75 ^th^ percentile for non-parametric quantitative values, and number and percentage for categorised values. The Student’s t-test was used for comparison between two independent mean groups for parametric data, and the Wilcoxon Rank Sum test was used for non-parametric quantitative data. The Chi-square test was used to study the association between each two variables or comparison between two independent groups as regards the categorized data. Pearson and Spearman correlation tests were used to study the correlation between parametric and non-parametric data respectively. The probability of error at 0.05 was considered significant, and 0.01 highly significant.

## RESULTS

The study included 30 patients, 11 males (36.67%) and 19 females (63.33%). Genders were confirmed by karyotype. The mean (SD) age of the patients was 10.22 (3.17) years. There were 23 (76.67%) patients with SW CAH and 7 (23.33%) were SV. Twenty healthy age- and sex-matched children were included as controls, 8 males (40%) and 12 females (60%). The mean (SD) age of the controls was 11.01 (2.71) years. Most (66.7%, 20 patients) were from a middle SES, 26.7% (8 patients) were of high SES, and 6.6% (2 patients) of low SES. Seventy per cent of the controls (14 subjects) were of middle SES, 25% (5 subjects) were of high SES, and 5% (1 subject) of low SES. No significant difference in SES was found between both groups ( *p* > 0.05). Sexual ambiguity at birth was evident in 19 patients (63.33%). Positive family history of CAH was present in 14 patients (46.7%). Some background data of the patients are listed in Tables 1 and 2 .

**Table 1:** Background data of patients

	Median (IQR)
Age at diagnosis (years)	0.34 (0.9)
Duration of treatment (years)	9.33 (5.77)
Hydrocortisone dose mg/m ^2^ /day	10 (7.63)
Total hydrocortisone dose (mg)	3585 (49923.75)
Fludrocortisone dose mg/day	0.08 (0.05)

**Table 2:** Comparison of anthropometric parameters in patients and controls

	Patients (n = 30) Mean ± SD	Controls (n = 20) Mean ± SD	p
Weight SDS	1.23 ± 0.95	0.96 ± 0.92	0.337
Height SDS	0.53 ± 1.54	-0.21 ± 1.49	0.101
BMI SDS	1.46 ± 0.92	1.6 ± 0.58	0.522

Patients performed worse than controls in verbal, performance, and total IQ categories of the WISC test ( *p* < 0.05) ( Table 3 ). This was also evident in the Benton Visual Retention test. Patients had significantly higher median difference between obtained and expected errors, and significantly lower obtained correct scores and higher expected error score ( *p* < 0.05) ( Table 4 ). In the Wisconsin Card Sorting Test, patients performed significantly worse compared to controls, with less correct trials, more errors, and more failed trials to complete the test categories ( *p* < 0.05) ( Table 5 ). When patients were classified according to their daily hydrocortisone doses (> 15, 10-15, and < 10 mg/m ^2^ /day), regarding the dose that was taken at the time of assessment, there were no significant differences detected in age, age at diagnosis, duration of treatment, and SES between the three groups ( *p* > 0.05). Height SDS, weight SDS, BMI SDS, and free testosterone level did not differ significantly ( *p* > 0.05). The WISC test, Benton Test and WCST results also did not significantly differ ( *p* > 0.05).

**Table 3:** Wechsler Intelligence Scale for Children (WISC) in patients and controls

	Patients n = 30 Median (25 ^th^ – 75 ^th^ )	Controls n = 20 Median (25 ^th^ – 75 ^th^ )	p
VIQ	85.5 (80-92.5)	109 (99-115.3)	0.000
Verbal comprehension	9 (7.8-10)	11.5 (10-13.8)	0.000
Verbal arithmetic	7 (5-9)	10.5 (7.3-12)	0.000
Verbal similarities	6 (5-9)	10 (9-12)	0.000
Verbal digit span	5 (4-6)	9 (8-10)	0.000
PIQ	80.5 (74-88.8)	105 (98.5-109)	0.000
Picture completion	7 (6-8)	10 (9-11)	0.000
Block design	6 (5-8)	9 (8-10)	0.000
Coding	6.5 (5-9)	11.5 (9-13)	0.000
TIQ	82.5 (75.8-90.3)	106 (100.3-111.8)	0.000

VIQ: verbal intelligence quotient, PIQ: performance intelligence quotient, TIQ: total intelligence quotient.

**Table 4:** Benton Visual Retention Test in patients and controls

	Patients n = 30 Median (25 ^th^ – 75 ^th^ )	Controls n = 20 Median (25 ^th^ – 75 ^th^ )	p
OCS	2 (1-4.3)	5.5 (4-7.8)	0.000
ECS	4 (2-6)	5 (4-6.8)	0.07
DIFF1	2 (0-2.3)	1 (0-2)	0.109
OES	12.5 (8.8-16)	6.5 (3.5-10)	0.000
EES	9 (6-12)	7 (4-8.8)	0.026
DIFF2	3.5 (2-6.3)	1 (1-2)	0.000

OCS: obtained correct score, ECS: expected correct score, DIFF1: difference between obtained and expected correct score, OES: obtained error score, EES: expected error score, DIFF2: difference between obtained and expected error score.

**Table 5:** Wisconsin Card Sorting Test (WCST) in patients and controls

	Patients n = 30 Median (25 ^th^ – 75 ^th^ )	Controls n = 20 Median (25 ^th^ – 75 ^th^ )	p
Trials administered	128 (128-128)	99 (83-104)	0.000
Total correct trials	58.5 (47.3-75.3)	73 (67-80.3)	0.007
Total error	72 (51.1-80.8)	19 (16-24)	0.000
% Error	56 (40-67)	19 (18-26)	0.000
Perseverative responses	42.5 (27.5-81.8)	10 (8-15)	0.000
% Perseverative responses	39.5 (23.5-71.3)	10 (10-15)	0.000
Perseverative error	40 (26.8-74)	10 (8-12)	0.000
% Perseverative error	33.5 (22.8-58)	10 (10-12)	0.000
Non perseverative error	22.5 (7-24.3)	9 (8-12)	0.04
% Perseverative error	17.5 (6.8-19.3)	10 (9-12)	0.07
Conceptual level responses	37.5 (11.8-68.3)	64 (62-73.5)	0.001
% Conceptual level responses	29.5 (13.8-54.3)	75 (65-76)	0.000
Categories completed	2 (1-4)	6 (6-6)	0.000
Trials to complete category	17.5 (12-76.5)	13 (11-13)	0.004
Failure to maintain set	1 (0-2)	0 (0-1)	0.056

Comparing patients and controls of the same SES showed that among those from high SES, patients had significantly worse PIQ and TIQ ( *p* < 0.05). Patients from high SES showed worse results than controls of the same SES in the difference between the obtained correct and expected correct scores in the Benton Visual Retention Test ( *p* < 0.05).

In the Wisconsin Card Sorting Test, patients of high SES did significantly worse in many aspects, which was particularly evident in the number of trials administered being highly significant among patients ( *p* < 0.01), and the number of completed test categories. ( *p* <0.05), ( Table 6 ).

**Table 6:** Cognitive tests among patients and controls of high SES

	Patients n = 8 Median (25 ^th^ – 75 ^th^ )	Controls n = 5 Median (25 ^th^ – 75 ^th^ )	p
**WISC**			
VIQ	96.5 (93-105.8)	104 (98.5-113)	0.163
PIQ	91.5 (81-98)	105 (99.5-109)	0.018
TIQ	93.5 (82-101.8)	106 (99.5-109.5)	0.048
**Benton Visual Retention Test**			
OCS	5.5 (1.8-6.8)	5 (3.5-6)	0.076
ECS	5.5 (3-7)	4 (4-6.5)	1
DIFF1	2 (1.3-3)	1 (0-1)	0.029
OES	7.5 (4.3-12.8)	9 (5-10.5)	0.712
EES	6.5 (5-10.8)	8 (5-8.5)	0.825
DIFF2	2.5 (1-4.5)	1 (1-2)	0.149
**Wisconsin Card Sorting Test**			
Trials administered	128 (115.3-128)	83 (76-101.5)	0.006
Categories completed	4 (2.3-5.8)	6 (6-6)	0.017

VIQ: verbal intelligence quotient, PIQ: performance intelligence quotient, TIQ: total intelligence quotient. OCS: obtained correct score, ECS: expected correct score, DIFF1: difference between obtained and expected correct score, OES: obtained error score, EES: expected error score, DIFF2: difference between obtained and expected error score.

Among patients and controls of middle SES, the VIQ, PIQ, and TIQ were highly significantly worse among patients ( *p* < 0.01). A highly significant difference was obtained between the obtained error and expected error score in the Benton Visual Retention Test showing worse performance among patients ( *p* < 0.01). In the Wisconsin Card Sorting Test, patients performed worse than controls in many aspects with a highly significant difference in the number of trials administered, and in the number of completed test categories ( *p* < 0.01), as well as a significant difference in the number of trials to complete test categories ( *p* < 0.05), ( Table 7 ). No significant differences existed in age or anthropometric data between patients and controls of high SES, or those of medium SES. We could not compare patients and controls of low SES due to the small sample size (2 and 1 respectively).

**Table 7:** Cognitive tests among patients and controls of medium SES

	Patients n = 20 Median (25 ^th^ – 75 ^th^ )	Controls n = 14 Median (25 ^th^ – 75 ^th^ )	p
**WISC**			
VIQ	85 (80-89.3)	110.5 (99-1166.5)	0.000
PIQ	80 (74-87)	105 (99.5-109)	0.000
TIQ	81 (75.3-85.5)	106.5 (100.8-112.5)	0.000
**Benton Visual Retention Test**			
OCS	2 (1-3.75)	6.5 (3.8-8)	0.000
ECS	3 (2-5.8)	5 (4-8)	0.043
DIFF1	1 (0-2)	1 (0-2)	0.432
OES	14.5 (9-16)	6 (3-10)	0.000
EES	10.5 (6.3-12)	7 (3.8-8.3)	0.02
DIFF2	4 (2-7)	1.5 (0-2)	0.001
**Wisconsin Card Sorting Test**			
Trials administered	128 (128-128)	99 (83-104.3)	0.000
Categories completed	1.5 (0.3-3.5)	6 (6-6)	0.000
Trials to complete	23.5 (13.3 – 116.3)	13 (11-14)	0.014

VIQ: verbal intelligence quotient, PIQ: performance intelligence quotient, TIQ: total intelligence quotient. OCS: obtained correct score, ECS: expected correct score, DIFF1: difference between obtained and expected correct score, OES: obtained error score, EES: expected error score, DIFF2: difference between obtained and expected error score.

**Figure 1:**
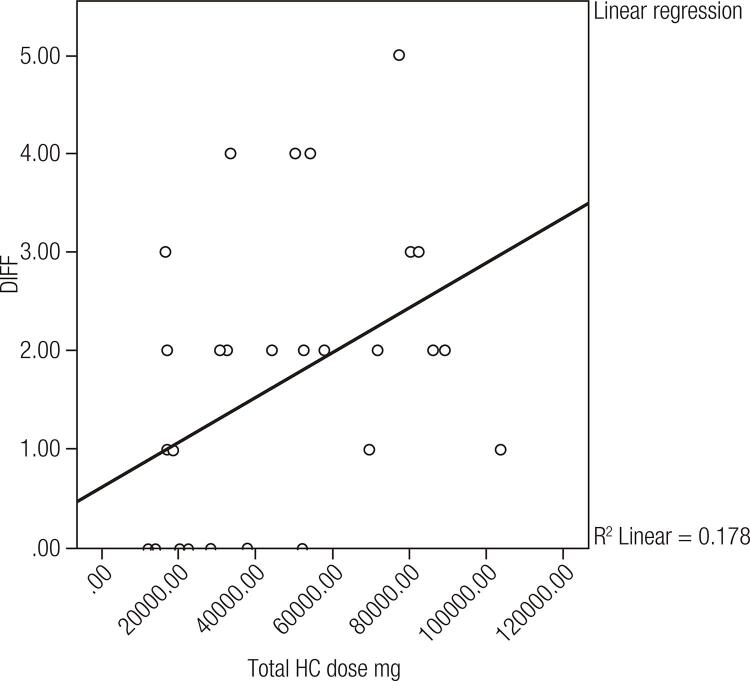
Correlation between Benton Visual Retention Test and Total HC dose ( *p* < 0.05).

The difference between expected and obtained error score (DIFF 2) of the Benton Retention Test showed a significant positive correlation with the total hydrocortisone dose in the patient group (r = 0.421, ( *p* < 0.05) ( Figure 1 ). Free testosterone did not significantly correlate with any of the performed tests ( *p* > 0.05).

## DISCUSSION

This study is one of the few studies addressing cognitive functions in children and adolescents in CAH. The present study shows that young patients with CAH have intellectual disabilities as reflected by lower total, verbal, and performance IQ scores. Furthermore, we find that these patients have impairment in both the visual memory and cortical executive functions. To our knowledge, this is one of the first studies to use the Benton Visual Retention Test and the Wisconsin Card Sorting Test in this population of patients. Although some of the patients’ scores fell within the normal ranges, they were still significantly lower than the scores obtained from healthy controls. There were no differences in SES between patients and controls sufficient to eliminate the effect of SES. Additionally, when patients and controls of the same SES were compared, patients still scored worse than controls. The strong family history of CAH in patients was related the fact that our clinic is a tertiary referral centre where we treat patients and some of their siblings as well. However, only one child from each family was included in the study to avoid overrepresentation of the genetic and environmental contribution to cognition in our studied sample.

Previous studies looking at the cognitive and psychosocial characteristics of patients with CAH showed controversial results ( 3 ). It is worth noting that most of these studies were conducted on older groups of patients. It was previously suggested that patients with CAH may have higher IQ scores. But when such patients were compared with their non-affected siblings, the difference in the IQ was not observed ( 19 , 20 ). The outdated tests used in these studies, the under-presentation of SW patients, over-representation of females, might all have been reasons for the observed results ( 8 ). More recent studies found lower IQ scores among patients with CAH ( 8 , 11 , 21 ). Collaer and cols. previously demonstrated lower scores in tasks examining spatial perception and short term memory. However, their cohort had a similar IQ to the controls ( 21 ), whereas the total IQ score in our patients was significantly lower than controls. Reduced verbal performance, irrespective of sex, was previously reported in CAH children ( 11 ).

Various factors could explain this: reduced endogenous glucocorticoid production, disruption of the hypothalamo-pituitary-adrenal axis, subsequent excess CRH and ACTH could all be the reason ( 22 ). Difficulties in adjusting glucocorticoid dose is another problem – it was demonstrated recently that excess glucocorticoid treatment increased pro-apoptotic signals in hippocampal neurons ( 23 ). The hippocampus plays a pivotal role in learning and memory, has high concentration of corticosteroid receptors ( 24 ), and is liable to damage by excess glucocorticoids. Some adults with CAH showed hippocampal dysgenesis ( 25 ). A recent study in adolescents and adults with CAH showed impaired executive functions represented by defects in visual and visuospatial working memory ( 26 ). Another recent study in adults showed that the doses of glucocorticoids that are used for treatment of CAH can alter brain micro- and macrostructure with quantitative brain magnetic resonance imaging (MRI) changes of reduced volume in the right hippocampus, thalami, cerebellum and brain stem. The effects of glucocorticoid treatment also affected the brain choline content of the mesial temporal lobe. Their patients also demonstrated cognitive impairment in some domains, e.g. working memory ( 27 ).

In this study, the total hydrocortisone dose correlated positively with the Benton Visual Retention Test DIFF2 result. This could possibly point to the deleterious effect of higher hydrocortisone dose on visual memory. The higher the cumulative hydrocortisone dose in our patients, the greater the difference there was between their obtained errors in the test and what was expected for their age. Such a finding was not observed when patients were compared according to the mean daily hydrocortisone dose. A previous study demonstrated that acute glucocorticoid administration negatively affects the different components of working memory, including executive functions ( 28 ). The positive correlation found in our study between total hydrocortisone dose and the Benton score supports this. Browne and cols. demonstrated reduced working memory in children with CAH, but the Digit Span test was used in their study ( 11 ). The negative effects of corticosteroids on learning and memory have been suggested using variable tests ( 29 - 31 ). These findings may call for a re-examination of the prescribed glucocorticoid dose in children with CAH to minimise short term memory deficits, which may affect their learning abilities, while still maintaining control over the excess intermediary metabolites of the cortisol synthesis pathway. Research in adult rats demonstrated that both high and low doses of glucocorticoids impaired memory ( 32 ). The same can occur in patients with CAH who are subjected to over- and under-dosing ( 33 ). There has been less research exploring a potential relationship in humans between low glucocorticoid levels and memory functioning, although an inverted U-shaped relationship between glucocorticoids and human memory retrieval has been demonstrated ( 34 ).

Antenatal and early postnatal exposure to high androgen levels is another possible explanation. Excess androgen exposure starting in utero was suggested to adversely affect the maturation of cerebral hemispheres, and to cause learning difficulties ( 8 ). Enhanced spatial abilities were previously observed in females with CAH compared to normal males, and on the other hand, males with idiopathic hypogonatropic hypogonadism showed lower spatial abilities ( 35 ). In our study, free testosterone levels did not correlate with the total IQ in the WISC test or the DIFF-2 of the Benton test, neither with any other test domain. Thus we could not prove that postnatal exposure to high androgen levels in children with CAH affects cognitive functions or spatial cognition, unlike that demonstrated in previous studies ( 5 ), ( 10 ). This may be due to differences in study designs, or differences in the age and sex of the included patients in previous studies.

Our cohort of CAH patients were not diagnosed by neonatal screening as it was not applied on a wide scale at the time of their diagnosis, and neither of them received prenatal dexamethasone, so the results on cognition bear no relation to prenatal dexamethasone administration.

Hyponatraemia is one of the mechanisms that may be incriminated in worse cognitive performance ( 8 ). We could not compare SW and SV patients due to the small number of the SV group. Some authors found that patients with salt-wasting CAH have lower overall cognitive ability than patients with the simple virilising form, but both groups were within the normal range ( 3 ). This finding was significant in women with CAH, but the effect was not present for the performance IQ ( 8 ). Conversely, adolescent and young adult SW females were found to perform better than SV females in mental rotation tasks, an effect that was not demonstrated in verbal fluency or perceptual speed. Furthermore, this enhanced performance was predicted by the severity of prenatal androgen exposure ( 36 ). Such controversies in this respect warrant further research.

The limitations of this study include the small sample size, which also hampered comparing salt wasters with simple virilisers. It would be useful to study the relation between hyponatraemia and cognitive performance in this specific group. This was also demonstrated in the worse performance in some of the cognitive tests in patients with a high socioeconomic status compared to controls, a finding which appears counterintuitive and contradictory to what is known in normal children. This effect of socioeconomic status on cognitive functions in children with CAH needs to be addressed in larger studies. One of the factors affecting cognition could be the severity of neonatal dehydration at first presentation, which is difficult to document in retrospect. Another limitation was the difficulty finding a larger comparison group, which is often a problem when assessing psychological problems in a young population with chronic illness. One cannot ignore the possible effect of the chronic illness itself on performance.

In conclusion, this study showed that impaired cognitive functions exist in children with CAH mainly in the forms of lower intellectual performance and worse visual memory as well as worse executive functions. The cumulative hydrocortisone dose is the most significant contributing factor in this regard, with an effect that seems to start early. We believe that further studies are warranted on greater numbers of patients. Specific neuro-imaging techniques are helpful in this regard. Early intervention in children with cognitive deficits is crucial to prevent learning deficits later in childhood.
